# The Phenotypic Characterization of the Human Renal Mononuclear Phagocytes Reveal a Co-Ordinated Response to Injury

**DOI:** 10.1371/journal.pone.0151674

**Published:** 2016-03-21

**Authors:** Dario A. Leone, Nicolas Kozakowski, Christoph Kornauth, Theresa Waidacher, Barbara Neudert, Agnes G. Loeffler, Andrea Haitel, Andrew J. Rees, Renate Kain

**Affiliations:** 1 Clinical Institute of Pathology, Medical University of Vienna, Vienna, Austria; 2 Surgical Pathology, University of Wisconsin, Madison, WI, United States of America; INSERM, FRANCE

## Abstract

Mammalian tissues contain networks of mononuclear phagocytes (MPh) that sense injury and orchestrate the response to it. In mice, this is affected by distinct populations of dendritic cells (DC), monocytes and macrophages and recent studies suggest the same is true for human skin and intestine but little is known about the kidney. Here we describe the analysis of MPh populations in five human kidneys and show they are highly heterogeneous and contain discrete populations of DC, monocytes and macrophages. These include: plasmacytoid DC (CD303^+^) and both types of conventional DC—cDC1 (CD141^+^ cells) and CD2 (CD1c^+^ cells); classical, non-classical and intermediate monocytes; and macrophages including a novel population of CD141^+^ macrophages clearly distinguishable from cDC1 cells. The relative size of the MPh populations differed between kidneys: the pDC population was bi-modally distributed being less than 2% of DC in two kidneys without severe injury and over 35% in the remaining three with low grade injury in the absence of morphological evidence of inflammation. There were profound differences in the other MPh populations in kidneys with high and low numbers of pDC. Thus, cDC1 cells were abundant (55 and 52.3%) when pDC were sparse and sparse (12.8–12.5%) when pDC were abundant, whereas the proportions of cDC2 cells and classical monocytes increased slightly in pDC high kidneys. We conclude that MPh are highly heterogeneous in human kidneys and that pDC infiltration indicative of low-grade injury does not occur in isolation but is part of a co-ordinated response affecting all renal DC, monocyte and macrophage populations.

## Introduction

Mammalian tissues contain networks of mononuclear phagocytes (MPh) that are critical for tissue homeostasis including sensing injury and promoting repair. The networks are especially prominent in organs in which epithelial surfaces are exposed to microorganisms or xenobiotics. For example, MPh in the kidney envelope the renal tubules and peritubular capillaries and extend processes into their lumens and so are ideally placed to co-ordinate immune responses to blood-borne and filtered antigens [1, 2]. Renal MPh also modulate renal inflammation and either cause injury or attenuate it depending on the setting [3–6]. However, the cues that determine these outcomes and the precise nature of the cells responsible remain unclear. Even the nature of MPh in the kidney and elsewhere was controversial since they reported to have the characteristics of both macrophages and dendritic cells (DC) [7, 8]. These controversies have been largely resolved by developments in cell lineage tracing in mice and transcriptomic and flow cytometric analyses that reveal tissue MPh contain multiple different cell types [9, 10].

In mice tissue MPh originate from three sources: embryonic macrophage-like cells that populate tissues during development and are maintained into adulthood by local cell division; circulating monocytes that infiltrate tissues and can mature into macrophages (and possibly DC) [11, 12]; and a unique bone marrow DC precursor that gives rise to plasmacytoid DC (pDC) and two types of conventional (or classical) DC (cDC1 and cDC2) that populate the tissues via the circulation [13]. Plasmacytoid DC are identified by expression of B220 in mice and CD303 in man and are rare in healthy organs but abundant after injury when they are the major source of type 1 interferon. By contrast both types of cDC are abundant in healthy tissue and functionally distinct: the cDC1 population express CD8 and CD103 in mice and CD141^+^ in man, and migrate from tissue to lymph nodes and present antigen to CD4 T cells [14, 15]; whereas cDC2 population express CD4 and CD11b in mice and CD1c in man, and are less migratory and have a unique role in presentation of glycolipid antigens and possibly antigens that evoke Th2 responses [16]. Dendritic cells, monocytes and macrophages and their subtypes have cell specific transcriptomic patterns [17, 18] shared by mice and men. This has led to the development of a common nomenclature for different types of MPh and their subtypes [19].

Mononuclear phagocytes were first identified in normal rodent and human kidneys by immunohistology using the monoclonal antibody, F4/80, and antibodies specific for MHC class II and CD11b, and shown to be more abundant after injury [20]. Analysis of mouse kidneys by flow cytometry showed that the predominant type of MPh in the cortex co-expressed CD11b and CD11c and functional studies confirmed they were able to present and cross present antigen [21]; this identifies them as authentic DC that would now be classified as the cDC2 subtype. The renal cortex contained a second less abundant population of what would now by called as cDC1 cells that expressed CD11c and CD103 but not CD11b [22]. Indeed, differential patterns of CD11b and CD11c expression have been used to resolve mouse renal MPh into at least five populations that include macrophages as well as DC [23]. The functional difference between the cDC subpopulations has been demonstrated in rat renal allografts which show that the cDC1 cells rapidly migrate out of the graft whereas the cDC2 cells do not [24].

Necessarily, characterization of MPh in human kidneys is largely confined to immunohistology. The MPh were originally described as DC on the basis of HLA-DR expression [25] and this interpretation has been supported by later studies that show the MPh also expressed the cDC2 cell marker CD1c sometimes together with DC-SIGN, a C-type lectin receptor up-regulated on immature DCs and the macrophage cell marker CD68 [26]. Cells expressing the pDC marker CD303 are rare in normal kidneys but abundant in disease [27, 28]. Clusters of DC-SIGN^+^/CD68^-^ cells and proliferating T-cell accumulate in some human renal transplants and have been associated with allograft rejection and a poor prognosis. The application of flow cytometry to human renal MPh has been limited to the analysis of DC by the gating strategy used and to the study of biopsies from individuals with progressive chronic kidney fibrosis [29]. All three human DC subsets were increased in the interstitial infiltrates from patients with chronic glomerular disease, and their number correlated with the extent of renal fibrosis [30]. However, tissue MPh are critical for tissue homeostasis as well as injury in mice and also in human skin and intestine [31, 32], but the renal MPh populations under homeostatic conditions remain to be characterized.

Here we have used flow cytometry to characterize the MPh in morphologically normal portions of human nephrectomy specimens using a gating strategy designed to capture monocytes and macrophages as well as DC. The results identify discrete populations of plasmacytoid and conventional DC; the three recognized subtypes of monocyte and macrophages maturing from them; and a previously unrecognized macrophage-like cell that expresses CD141 and CD11b but not CD11c. Despite normal morphology, some kidneys had low-grade inflammation indicated by infiltration with pDC. Increased abundance of pDC invariably correlated with consistent changes in the proportions of other MPh populations. Accordingly, our characterization of MPh in human kidneys has identified a co-ordinated response to injury that provides a new insight into homeostasis within the human kidney and its response to injury.

## Material and Methods

### Kidney samples

Samples of the renal cortex were taken from five human nephrectomy specimens: three radical tumor nephrectomies (RTN); two cadaveric donor nephrectomies that could not be transplanted for technical reasons (CDN); and one radical nephrectomy (RN) performed because of uncontrollable proteinuria in an individual with Finnish type nephrotic syndrome. The ages of the four remaining kidneys were comparable (69 and 71 years for the donor nephrectomies and 56 and 62 for the tumor nephrectomies) and this was reflected in their morphological appearances. None of the kidneys had evidence of glomerular or tubulointerstitial inflammation but the four from adult individuals had mild renovascular disease. This affected 5–10% of the cortical area and was associated with glomerular scars, focal tubular atrophy, interstitial fibrosis and mild to moderate arteriolosclerosis. There was little variation in leukocyte infiltration between these kidneys, assessed with by CD45 (leukocyte common antigen) staining. It was generally sparse, although more prominent where there was tubular atrophy.

The kidney samples were used to characterize renal MPh using a protocol based on one developed for mice [33]. A sample of 1cm^3^ of cortical tissue was removed from each kidney which in the case of the tumor nephrectomies was taken from the opposite pole to the tumor at least 2 cm from its margin. The samples were placed in 24 well tissue culture plates and injected with 10 μg/mL collagenase type IV (Worthington Biochemical Corporation) and 30 μg/mL DNase I (Roche) in 1mL PBS containing EDTA and 5% FCS. After 30 minutes of incubation in a 5% CO2 humidified incubator at 37°C, the tissue was homogenized with a pestle and incubated for a further 15 minutes at 37°C. In order to obtain a single cell suspension and separate the structural tissue from the immune cells, the homogenate was then layered onto a density gradient (1,077 g/mL) with Ficoll-Paque PLUS (GE Heathcare life sciences, Sweden) and centrifuged at 800g for 20 minutes at room temperature. This last step was selected among other separation methods, for example based on filtration because the layer obtained using ficoll density gradient is highly enriched with viable mononuclear cells, excluding dead cells, debris and also polymorphic nuclear cell and erythrocytes.

### FACS staining

All antibodies were diluted in 5 ml of standard FACS buffer (PBS, 2% FSC and EDTA). After blocking Fc receptors by incubating for 20 minutes with Fc blocking reagent (Miltenyi Biotec), the cells were separated into aliquots containing at least 5x10^6^ cells per tube and stained separately. Each aliquot was stained with a panel of antibodies or isotype matched controls for 30 minutes on ice. An antibody to CD45 conjugated with APC-Cy7 (1 mg/ml, 1:200), was used to identify leukocyte populations and separate them from remaining renal cells. In the case of CD68 the cells were fixed with 3.5% paraformaldehyde for 10 minutes at room temperature and then permeabilized using BD Perm/Wash™.

FACS acquisition was performed with BD LSR Fortessa equipped with 4 lasers (405nm, 488 nm, 561 nm, 640 nm) and at least 3x10^6^ events were recorded in order to detect rare cell populations. FlowJo software (Tree Star Inc, Ashland, USA) was used for FACS analysis and documentation. The isotype control antibody were matched to the host species and fluorophores used for the staining, and were used alone (i.e. for assign the CD45 gate) and in combination with our panel of staining antibodies used to identify different populations of MPh (for example, CD45 APC-Cy7 + mouse and anti-human isotype APC were used to assign the CD45^+^ CD11c^+^ APC population, S1 Fig)

### Antibodies

The following directly labeled antibodies were used for FACS analysis CD45 APC-Cy7 clone: H130 (1 mg/ml, 1:200), CD3 PE-Cy7 clone: UCHT1 (1 mg/ml, 1:100), HLA-DR PE clone: LN3 (1 mg/ml, 1:400), CD68 PerCp-Cy5.5 clone: RPA T8 (1 mg/ml, 1:50), CD303 APC clone: 201A (1 mg/ml, 1:100)–all from Biolegend; CD19 PerCp-Cy5.5 clone: HIB19 (1 mg/ml, 1:50), CD11c APC clone: Bu15 (1 mg/ml, 1:100), CD14 PE-cy7 clone: 61D3 (1 mg/ml, 1:200), CD16 PE clone: B73.1 (1 mg/ml, 1:200), CD1c FITC clone: L161 (1 mg/ml, 1:50), DC-SIGN PE-Cy7 clone: eB-h209 (1 mg/ml, 1:100)–all from ebioscience; CD11b Pacific blue clone: CBMR1/5 (1 mg/ml, 1:50) and CD11b Alexa 700 clone: CBMR1/5 (1 mg/ml, 1:300)–from BD bioscience; HLA-DR FITC clone: AC122 (1 mg/ml, 1:400) and CD141 PE clone: AD5-14H12 (1 mg/ml, 1:50)–from Miltenyi Biotec; and 25F9 FITC clone: HM2158F (1 mg/ml, 1:50) from Hycult.

### Immunohistology

Renal cortical tissue was taken from areas adjacent to those analysed by flow cytometry and examined by light microscopy and immunohistology. All the histological specimens were fixed in 7.5% buffered formaldehyde and embedded in paraffin. We stained 4μm sections with haematoxylin and eosin, methenamine silver, PAS (Periodic Acid Schiff) and AFOG (Acid Fuchsine OrangeG) whilst 2μm thick consecutive sections were stained with antibodies to CD45 (LCA ready to use, 2B11+PD7/26; Ventana 760–4279), CD1c (1:50, clone 5B8, ab139333; ABCAM) and CD141 (1:100, M0617, DAKO, Vienna, Austria) or CD303 (1:25, clone 108H10.03, ref. DDX0040, Dendritics, Lyon, France). In some experiments, we used sections for double staining with antibodies to CD141 or CD303 and CD68 (1:100, M0876, DAKO, Vienna, Austria). Binding of antibodies was visualised manually or in an automated staining system (BenchMark ULTRA, Ventana-Roche, Basel Switzerland) using either HRP (manual protocol; plus Alkaline Phosphosphatase for double staining; both: Ultravision polymer kit, Thermo Scientific) or DAB (automated protocol; Plus Alkaline Phosphatase Red for double staining; both: ultra View Universal Detection KIT, Ventana-Roche, Basel Switzerland) following the manufacturers’ instructions. Sections were analysed by three pathologists blinded to the nature of the specimen independently and images were acquired on Zeiss Scope A1 using Axio Cam ICc3.

### Ethical issues

The study was performed in accordance with the conditions of the Helsinki declaration. None of the patients had any intervention that was not required for clinical care and the protocol and all the procedures required were reviewed and approved by the Ethics committee of the Medical University of Vienna (Approval: 1046/2015).

Prior to surgery, the patients signed a general consent agreeing that histopathological samples and tissue that were surplus to diagnostic requirements could be used for research in studies agreed by Ethics committee of the Medical University of Vienna without further consent: this is in line with Austrian Law. Accordingly the Ethics committee of the Medical University of Vienna reviewed all the procedures required and granted approval for the study protocol without the additional need for consent from the individual patients or their family (Approval: 1046/2015).

The kidney samples used for FACS analysis of leukocytes in the study were portions of the nephrectomy specimens that were surplus to the requirements for pathological diagnosis and would otherwise have been destroyed.

The kidney samples were removed and anonymised by the clinical pathologists responsible for the pathological examination of the surgical specimen. They were necessarily aware of the names of the patients but were not involved in the conduct or supervision of flow cytometry studies. The anonymised samples were transferred to the scientists performing the analysis who had no knowledge of the identity of the patients they originated from or access to their clinical or demographic data. The entire portion of the sample was used to prepare the single cell preparations that were used to characterise the renal MPh and none was retained after the analysis. Pseudonymised histological specimen taken as part of routine histopathological examination were used in the study for immunohistochemistry with antibodies to leukocyte subsets and staining patterns were evaluated by three certified histopahtologists (AGL, NK, RK) blinded to the diagnosis or reason for nephrectomy. Only authorized persons involved in the management of the patients had access to the original data and the pseudonymous data were stored and evaluated in the Clinical Institute of Pathology in a PC with restricted access.

The entire kidney sample was used to prepare the single cell preparations that were used to characterise the renal MPh and none was retained after the analysis.

## Results

### Characterization of human mononuclear phagocytic system (MPS)

**Fig 1:**
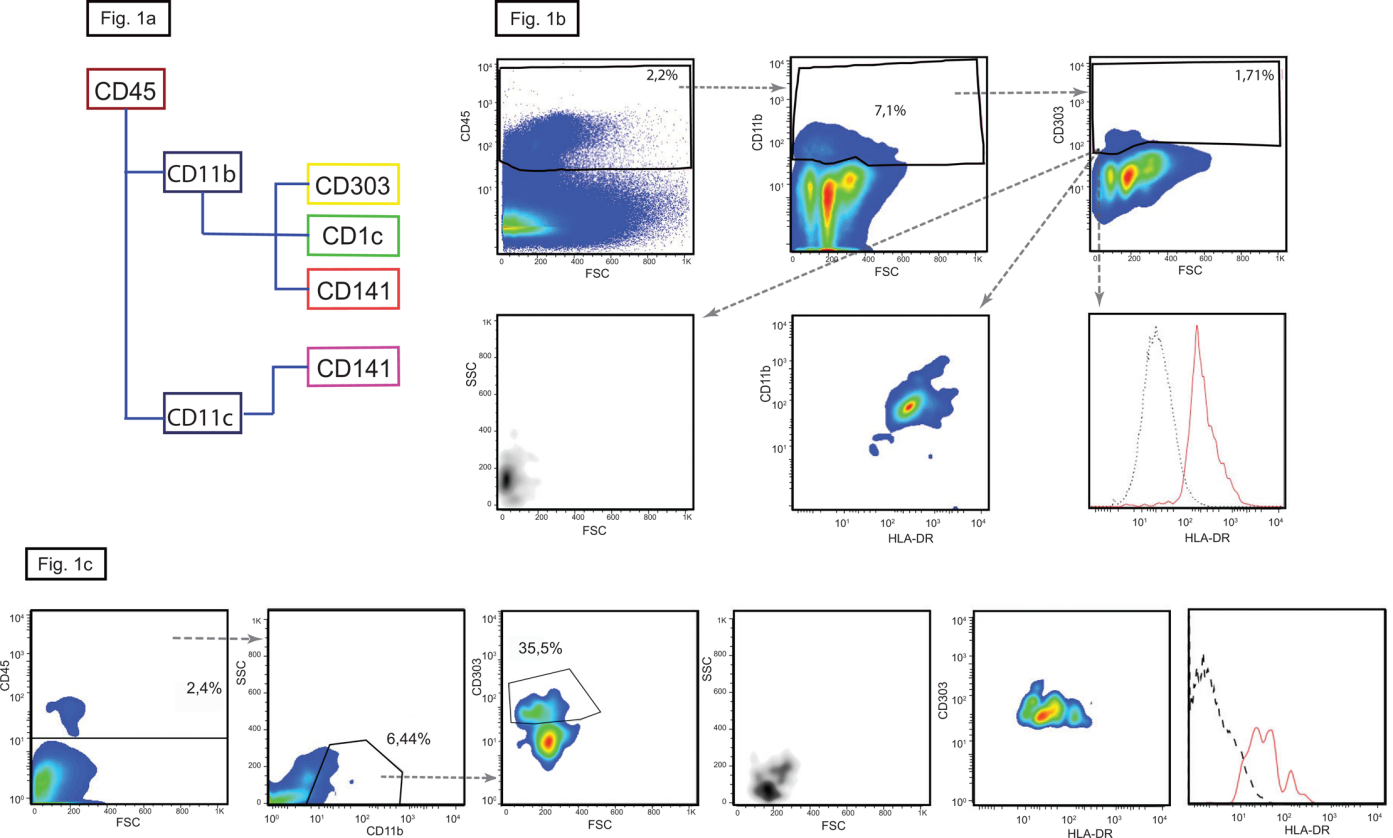
Plasmacytoid dendritic cells in hukman kidney. (a) Gating strategy with the markers used to differentiate between the pDC in yellow, cDC1 in purple and cDC2 in green. (b) CD303^+^ pDCs are less then 2% of the total CD11b^+^ cells (upper panels). Plasmacytoid DCs are small and round cells positive for HLA-DR (lower panels). In here the plots of the kidney n° 4 are shown, similar results were obtained with the kidney n° 5. (c) In here the plots of the kidney n°2 characterized by high numbers of infiltrating pDC are shown, similar results were obtained with the kidneys n° 1 and 3.

We characterized the renal MPh in single cell preparations from samples of five human kidneys. Of these, two were from cadaveric donor kidneys that could not be transplanted for technical reasons; two were from normal parts of kidneys removed because of a renal cell carcinoma; and one was from the kidney of an individual with congenital nephrotic syndrome. These were analysed using a gating strategy designed to capture all renal MPh rather than DC alone (Tables 1 and 2). First, we gated on CD45 positive cells staining the cells with antibodies to CD14 and the integrins CD11b and CD11c to identify all the myeloid cells within the leukocyte pool (Fig 1A). The myeloid cells were then stained with a panel of antibodies commonly used to identify single MPh populations. This strategy enabled us to identify the different monocyte and DC subtypes and to ascertain whether cellular expression of a particular marker was unique to a particular cell type or expressed in a broader range of cells.

**Table 1:** Antibody panels.

General immune system	CD45, CD3, CD19, HLA-DR, CD11b, CD11c
Monocytes/Macrophages	CD45, CD11c, CD11b, CD14, CD16, 25F9, CD68
Plasmacytoid DCs	CD45, CD11b, CD303, DC-SIGN, HLA-DR
cDC1	CD45, CD11b, CD11c, CD141, DC-SIGN, HLA-DR
cDC2	CD45, CD11b, CD11c, CD1c, DC-SIGN, HLA-DR

**Table 2:** Characterization of nephrectomy specimen used for isolation of infiltrating leukocytes and histological analysis.

	Age/Sex	Pathology
1 RTN	62 / male	TCC G3, pT3, pN0 / mild to moderate renovascular disease
2 RTN	56 / male	RCC (clear cell), G2, pT2, pNX / mild to moderate renovascular disease
3 RN	3,5 / male	Nephrotic syndrome Finnish type
4 CDN	71 / male	Suspicion of malignancy / mild to moderate renovascular disease
5 CDN	69 / male	Fibromatous plaque renal artery / mild to moderate renovascular disease

RTN: Radical tumour nephrectomy, TCC: Transitional cell carcinoma RN: Radical nephrectomy, CDN: Cadaveric donor nephrectomy. Cellular differentiation (G2 moderate, G3 poor). Staging: pTNM [ref: UICC TNM Classification of Malignant Tumours, 7^th^ Edition, 2009, Leslie H. Sobin (Editor), Mary K. Gospodarowicz (Editor), Christian Wittekind (Editor)

### Characterization of dendritic cells

In human blood, the three DC subsets are identified by their unique expression of specific markers: pDC—CD303; cDC1—CD141 and cDC2 -CD1c. All five kidneys contained mutually exclusive populations of myeloid cells expressing these molecules. The CD303 positive cells were small with low granularity and uniformly expressed CD11b and HLA-DR, thus identifying them as typical pDC. The proportion pDC varied greatly and was bi-modally distributed being less than 2% in two kidneys and over 35% in the remaining three, indicating low-grade inflammation despite apparently normal morphology (Table 3).

**Table 3:** Relative percentage of Dendritic cell subtype.

	1 RTN	2 RTN	3 RN	4 CDN	5 CDN
pDC	36.0%	35.5%	39.2%	1.7%	1.5%
cDC1	12.8%	12.5%	10.0%	55.0%	52.3%
cDC2	51.2%	52.0%	50.8%	43.3%	46.2%

**Fig 2:**
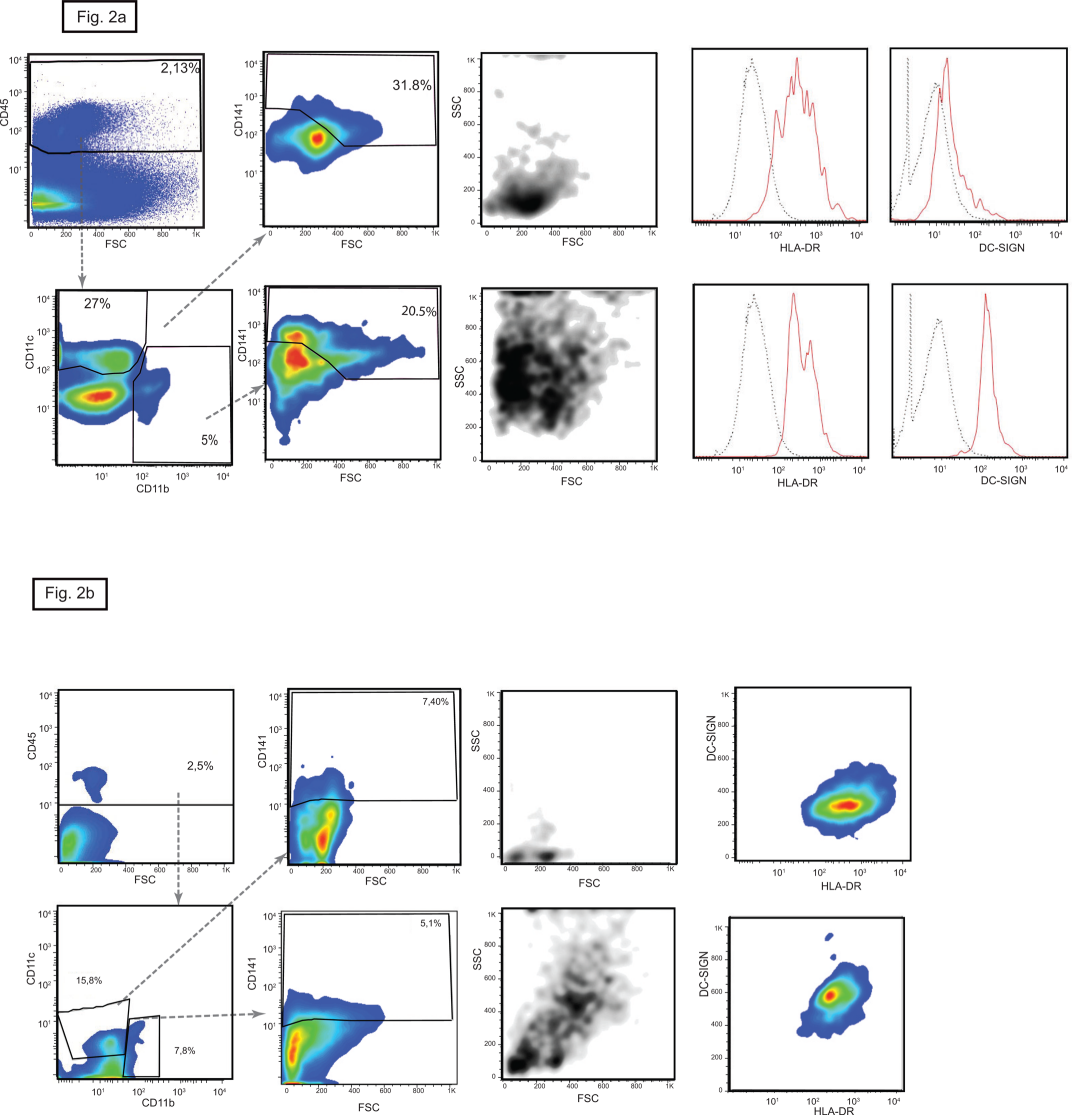
CD141 positive cells in human kidney. Two distinct populations of CD11c^+^ CD11b^-^ (upper panels) and CD11c^-^ CD11b^+^ (lower panels) express the cDC1 marker CD141. CD11c^+^ CD141^+^ cells have the typical dendritic cell shape and high expression of HLA-DR but are negative for DC-SIGN. These characteristics identify them as cDC1 cells. The CD11b^+^ CD141^+^ have similar granularity to macrophages and express DC-SIGN together with HLA-DR. In here the plots of the kidney n° 5 are shown, similar results were obtained with the kidney n° 4. (b) In here the CD141 positive cells, plots of the kidney n°2 characterized by high numbers of infiltrating pDC are shown similar results were obtained with the kidneys n° 1 and 3.

The proportions of CD141 expressing cells in the kidneys was also bi-modally distributed and correlated numerically inversely with the extent of the pDC infiltrate (Table 3). In blood, CD141 is expressed uniquely on cDC1 cells, but this was not the case in the kidney. Here, the CD141^+^ cells segregated into two subsets that differed in size and granularity, and in expression of CD11b and CD11c. Around 60% of the CD141^+^ cells were small with little granularity and expressed CD11c but not CD11b which identified them as cDC1 cells. The remaining 40% had the characteristics of macrophages: they were larger and more granular and expressed CD11b but not CD11c. Both these subsets expressed HLA-DR but DC-SIGN was confined to the CD11b^+^ macrophage-like cells (Fig 2).

**Fig 3:**
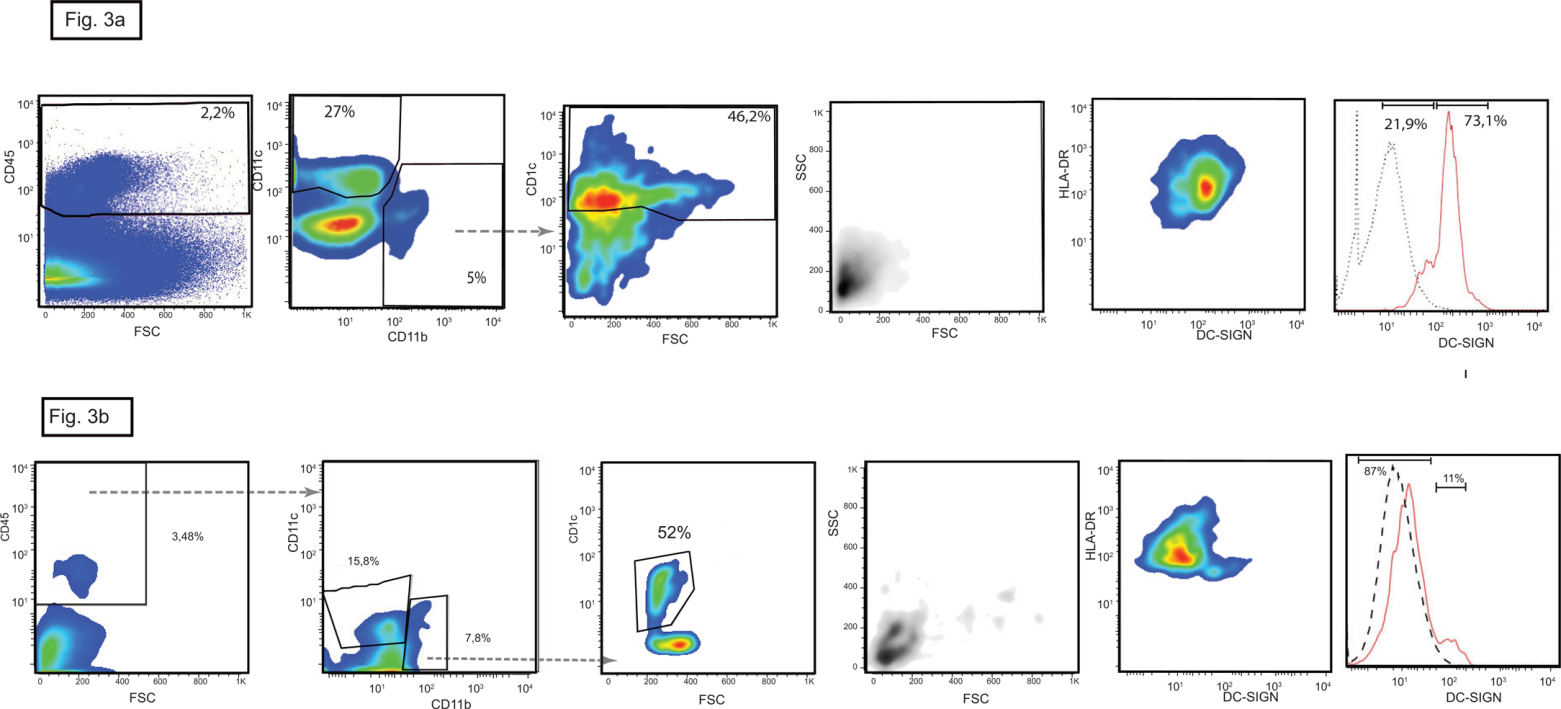
cDC2 dendritic cells in human kidney. The marker CD1c is expressed by CD11b^+^ cells (left panel) but not by CD11c^+^ cells. The cDC2 subtype has low granularity and size, all express HLA-DR and 73% express high levels of DC-SIGN. In here the plots of the kidney n° 5 are shown, similar results were obtained with the kidney n°4. (b) In here the CD1c positive cells, plots of the kidney n°2 characterized by high numbers of infiltrating pDC are shown, similar results were obtained with the kidneys n° 1 and 3.

There was a homogeneous population of CD1c^+^ cells that were small with low side scatter and uniformly expressed CD11b and HLA-DR and in 50–70% of cases also expressed high levels of DC-SIGN: this is consistent with the previously reported characteristics of cDC2 [27]. These cDC2 cells accounted for 43% to 52% of the total DCs population and so their proportions varied less than those of the other two DC subtypes (Fig 3).

Collectively, the results identify the three DC subtypes in human kidneys and show that expression of CD303, CD141 and CD1c discriminates between them. The CD303 and CD1c molecules are unique to their respective DC subset in the kidney whereas CD141 was expressed both by cDC1 cells and a population of macrophage like cells that are distinguished by their physical properties and the differential expression of CD11b and CD11c.

### Monocytes and macrophages

**Fig 4:**
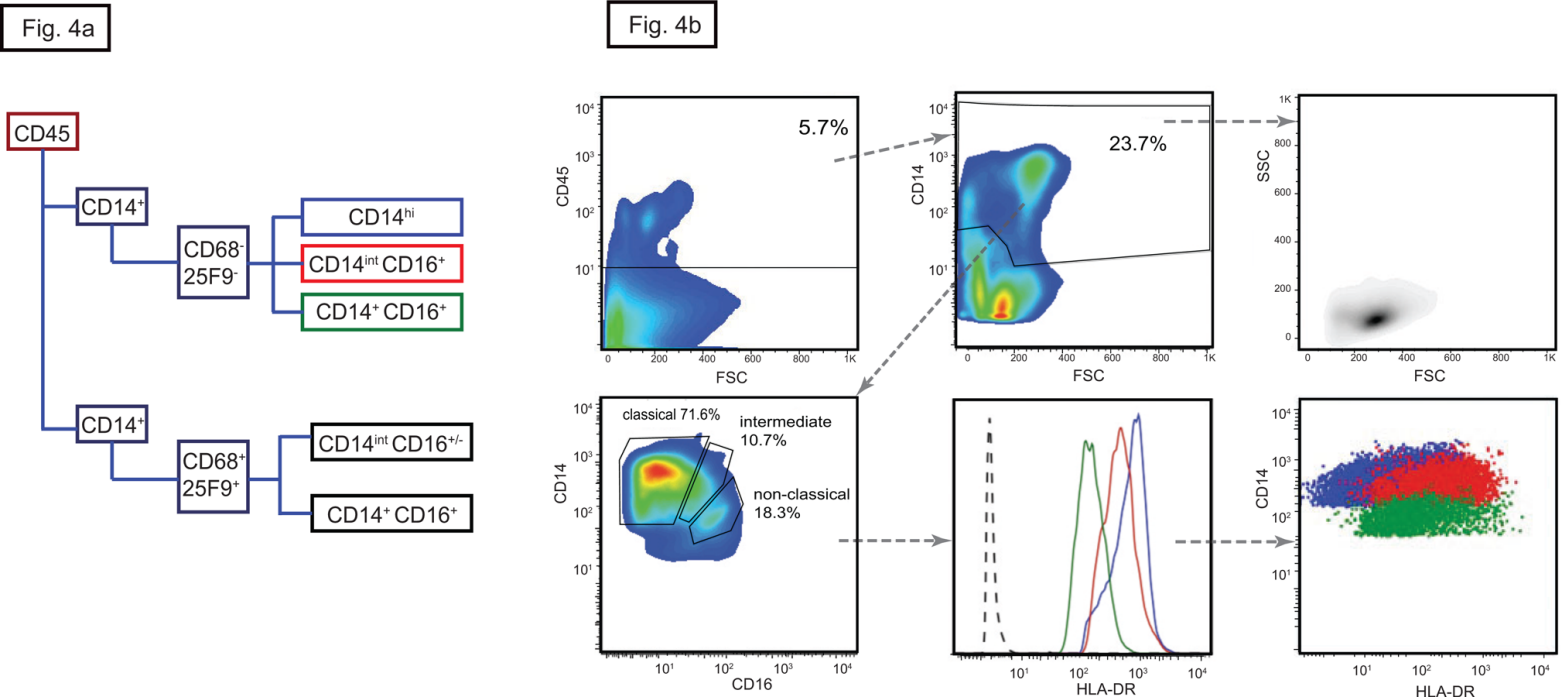
Monocytes in human kidney. (a) Gating strategy with the markers used to identify monocytes and macrophages. The boxes are color-coded with classical monocytes in blue, non-classical in green and intermediate in red. (b) Dot-plot of the CD45 gate followed by CD14 versus CD16 gate showing the relative percentage of classical, non-classical and intermediate monocytes (lower left). The monocytes were homogeneous in size and granularity (upper right). Differential expression of HLA-DR (lower central) can be use instead of CD16 to differentiate between the three subtypes which are distinguished by the same color coding as in 1a (lower right). In here the plots of the kidney n°5 are shown, similar results were obtained with kidney n°4.

**Fig 5:**
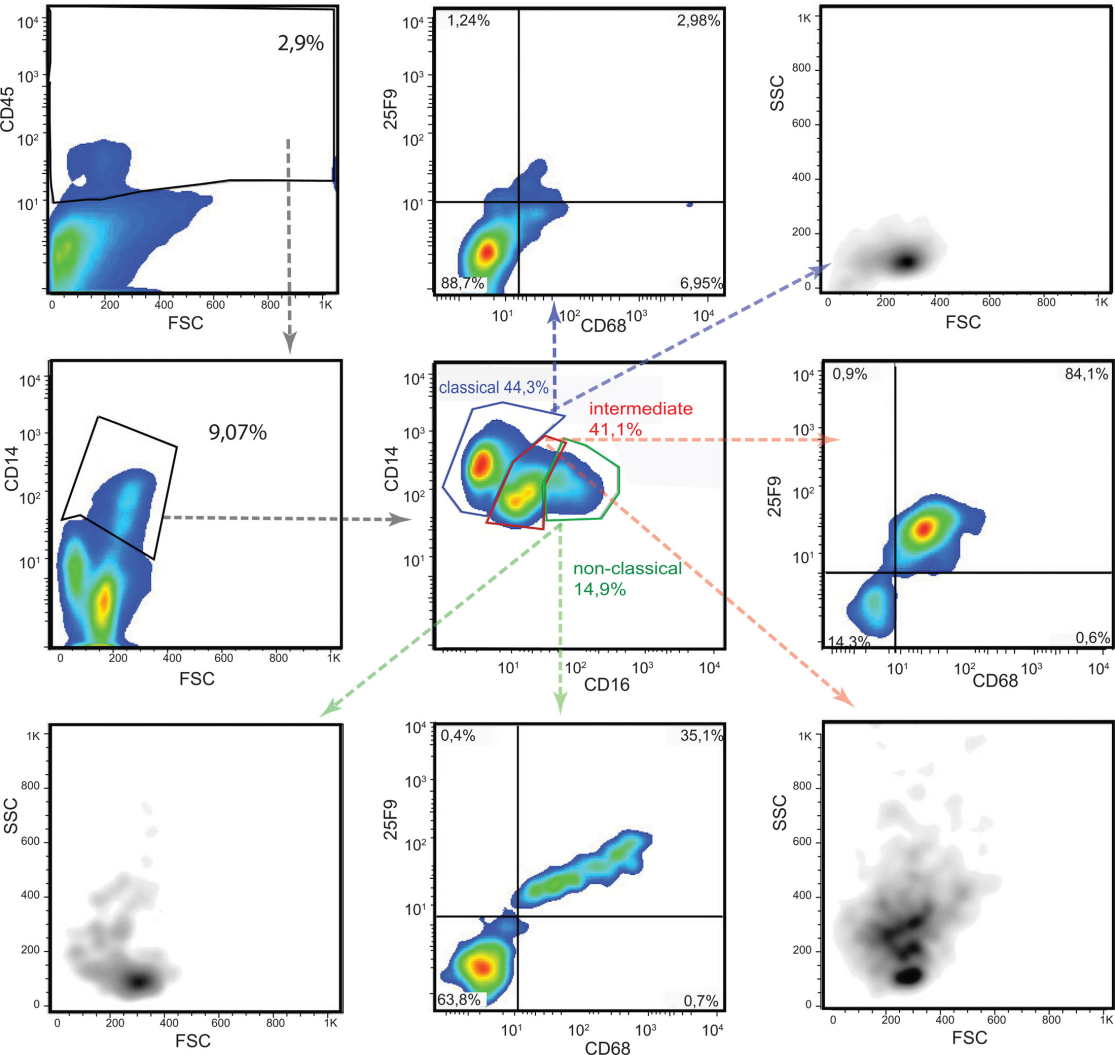
Monocytes derived macrophages in inflamed kidney. The proportion of CD14 positive cells that express CD16 cells is greater in kidneys with low numbers of pDC than in those with high numbers of pDC, and includes both CD14^int^ CD16^+^ and CD14^+^CD16^+^(central panel). The addition of 25F9, a marker for monocyte derived infiltrating macrophages and the active phagocytic marker CD68 reveals that the expanded population includes CD14 positive macrophages in addition to non-classical and intermediate monocytes, as it shown also by the scatter plot property. In here the plots of the kidney n° 2 are shown, similar results were obtained with the kidneys n° 1 and 3.

**Fig 6:**
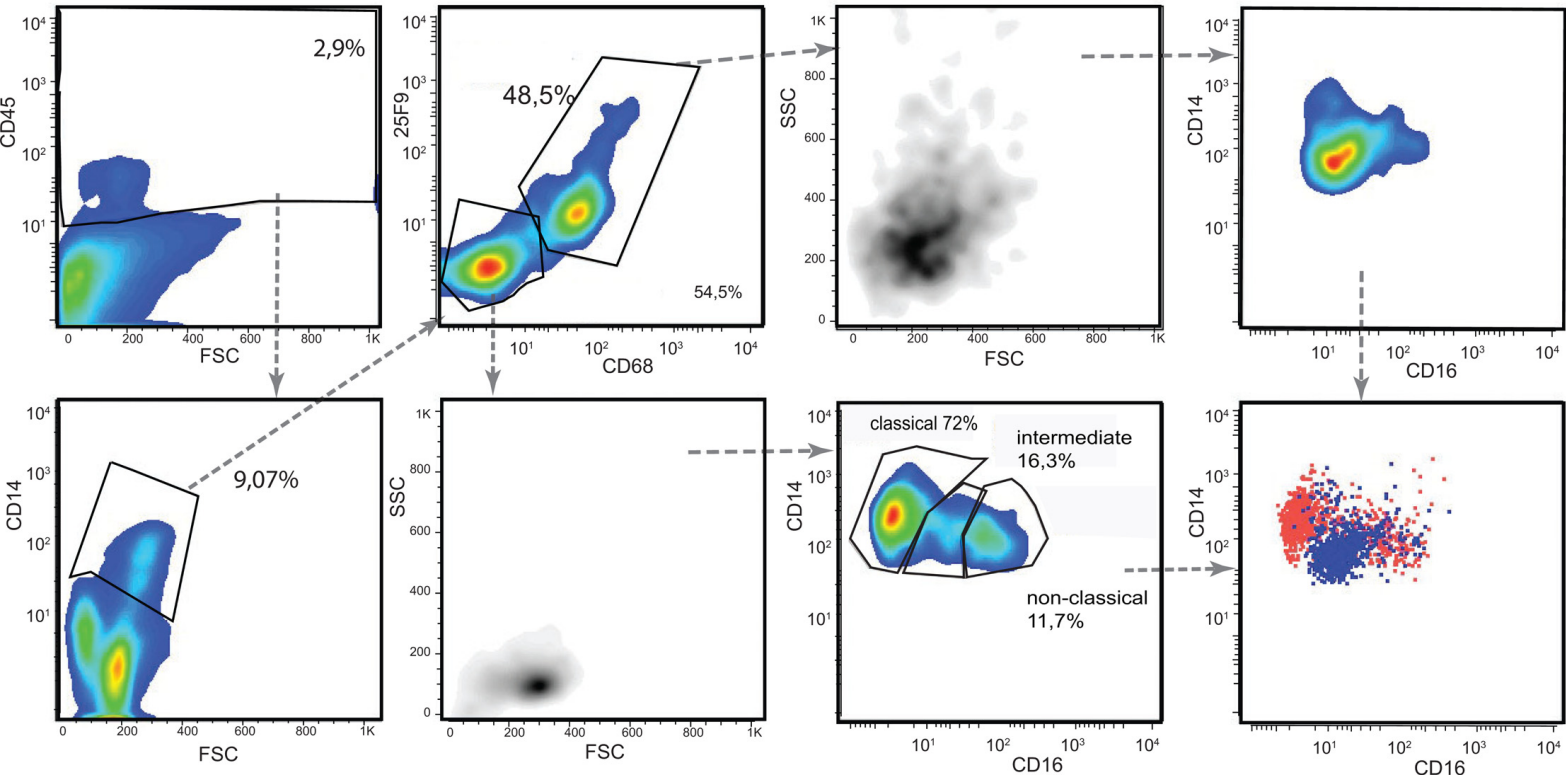
Gating out macrophages reconstitutes a monocyte percentage similar to the less inflamed kidney. Gating out the CD14^+^ CD68^+^25F9^+^ macrophages reveals similar proportions of the three monocyte subtypes as in the less inflamed kidney. The CD68^+^25F9^+^ have the shape of macrophages and they share similar levels of CD14 and CD16 in respect to intermediate and non-classical macrophages as indicated by the merge plot were blue dots are macrophages and red dots are monocytes (lower right). In here the plots of the kidney n° 2 are shown, similar results were obtained with the kidneys n° 1 and 3.

Human monocytes are segregated into three subtypes on the basis of CD14 and CD16 expression (Fig 4A): classical (CD14^high^ CD16^-^) monocytes that infiltrate tissues; non-classical (CD14^+^CD16^+^) monocytes that remain within the vasculature and patrol the endothelial surface; and intermediate (CD14^dim^ CD16^+^) monocytes which express the highest levels of HLA-DR but whose function is uncertain (Fig 4B) [34, 35]. All three subtypes were present within the renal MPh pool but with a relatively higher proportions of non-classical and intermediate monocytes than reported in blood (Fig 4B and Table 4). Resolution into the three subtypes was straightforward in kidneys with few pDC but more complex in those with abundant pDC (Fig 5). In these, most CD14 positive cells had high or intermediate levels of CD16 and many also expressed the macrophage markers 25F9 and CD68, molecules expressed on inflammatory macrophages with high phagocytic activity. However, gating out the CD68/25F9 double positive cells revealed the three underlying monocyte subpopulations in similar proportions to those found in the other kidneys (Fig 6). The abundance of CD68 and 25F9 on these cells correlated directly with the strength of expression of CD16, CD11b and CD11c and inversely with that of CD14 (not shown). The plots confirm that the CD68/25F9 negative cells are bona fide monocytes with low side scatter, whereas the CD68/25F9 positive cells have size and granular property of macrophages. Together, the data suggest this new population consists of infiltrating monocytes at various stages of maturation into macrophages

**Table 4:** Percentage monocytes subtype (Gated on 25F9^-^ CD68^-^) and Macrophages.

	1 RTN	2 RTN	3 RN	4 CDN	5 CDN
Classical CD14 ^hi^ CD16^-^	74.3%	72.0%	70.6%	70.0%	71.6%
Intermediate CD14^int^ CD16^+^	14.3%	16.3%	18.2%	12.6%	10.7%
Non-classical CD14^+^CD16	11.4%	11.7%	11.2%	17.4%	18.3%
Macrophages CD11b^+^25F9^+^	45.0%	48.5%	49.0%	19.2%	20.0%

### Analysis of renal MPh identifies a co-ordinated response to injury

**Fig 7:**
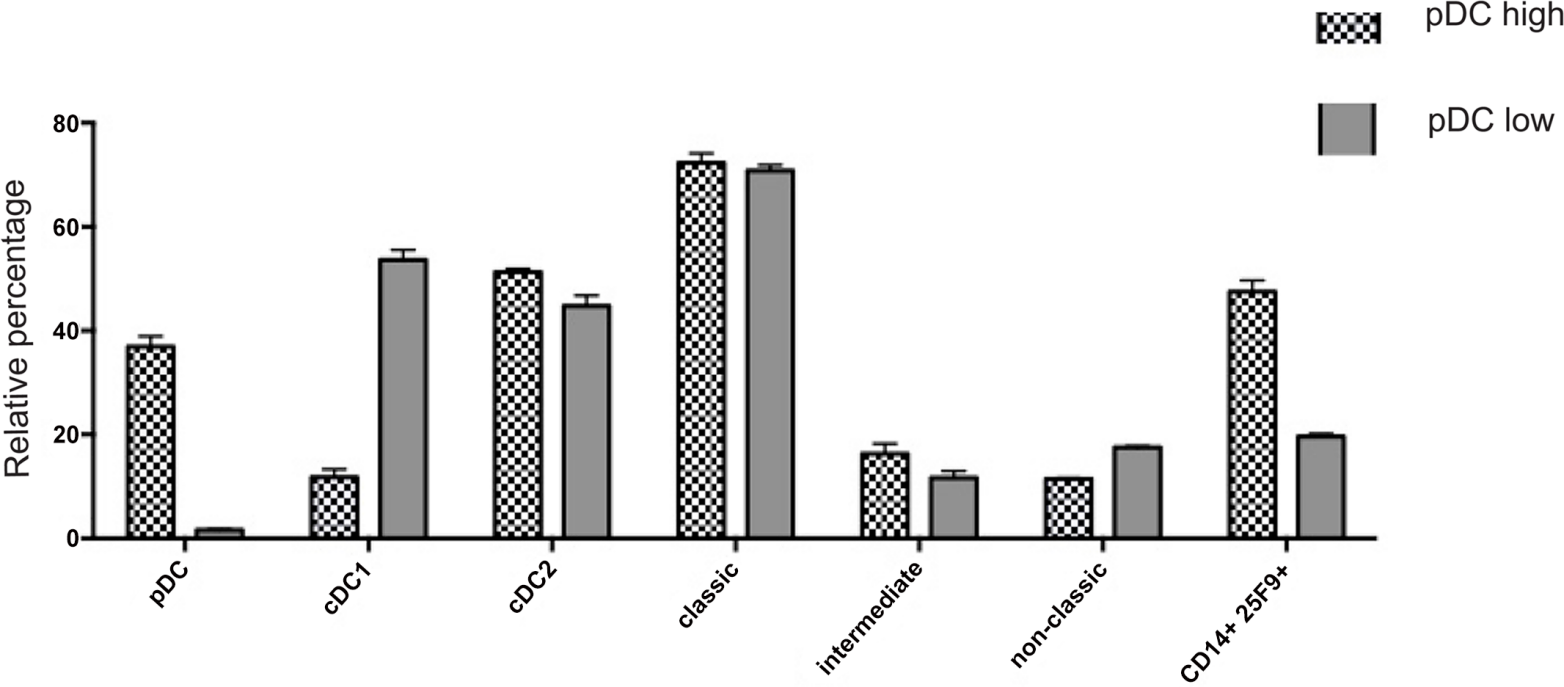
The correlation among the renal MPh suggests a co-ordinate response to inflammation. The relative percentage of the DC and monocytes subtypes and macrophages of the five kidneys clustered into two groups based on the level of pDC.

Immunohistological studies of the kidney and elsewhere have consistently shown that healthy tissue contains very small numbers of infiltrating pDC which increases rapidly after injury [26–28]. Plasmacytoid DC expressing CD303 were rare in both potential kidney donors who were without evidence of kidney disease whereas they were much more abundant in the remaining three kidney specimens, suggesting they had a degree of underlying injury. Two of these specimens were taken from apparently normal portions of radical nephrectomies containing a RCC, and one with congenital nephrotic syndrome. Separating the kidneys into two groups based on the relative abundance of pDC also segregated the cDC1 and macrophages into two non-overlapping groups (Fig 7). The kidneys with abundant pDC had a much lower proportion of CD141^+^ cells (12.8%, 12.5% and 10% of total DC) compared to the kidneys with sparse pDC (55% and 52.3% respectively) whereas there was no difference in the proportion of cDC2. This together with magnitude of the change excludes simple dilution by infiltrating pDC as an explanation for the proportionate reduction in CD141^+^ cells. The kidneys with abundant pDC also contained a population of CD14 positive cells that also expressed CD16 and the macrophage markers 25F9 and CD68 which were rare in kidneys with sparse pDC. Consequently, the cues responsible for recruiting pDC into the kidney also induce (or are associated with signals that induce) disappearance of cDC1 cells and the maturation of CD14 positive cells into macrophages confirming that pDC infiltration is part of a co-ordinated renal MPh response to injury.

### Immunohistological characterization

Next we determined whether the changes in the proportions of DC subsets identified by flow cytometry could be visualized immunohistochemically.

**Fig 8:**
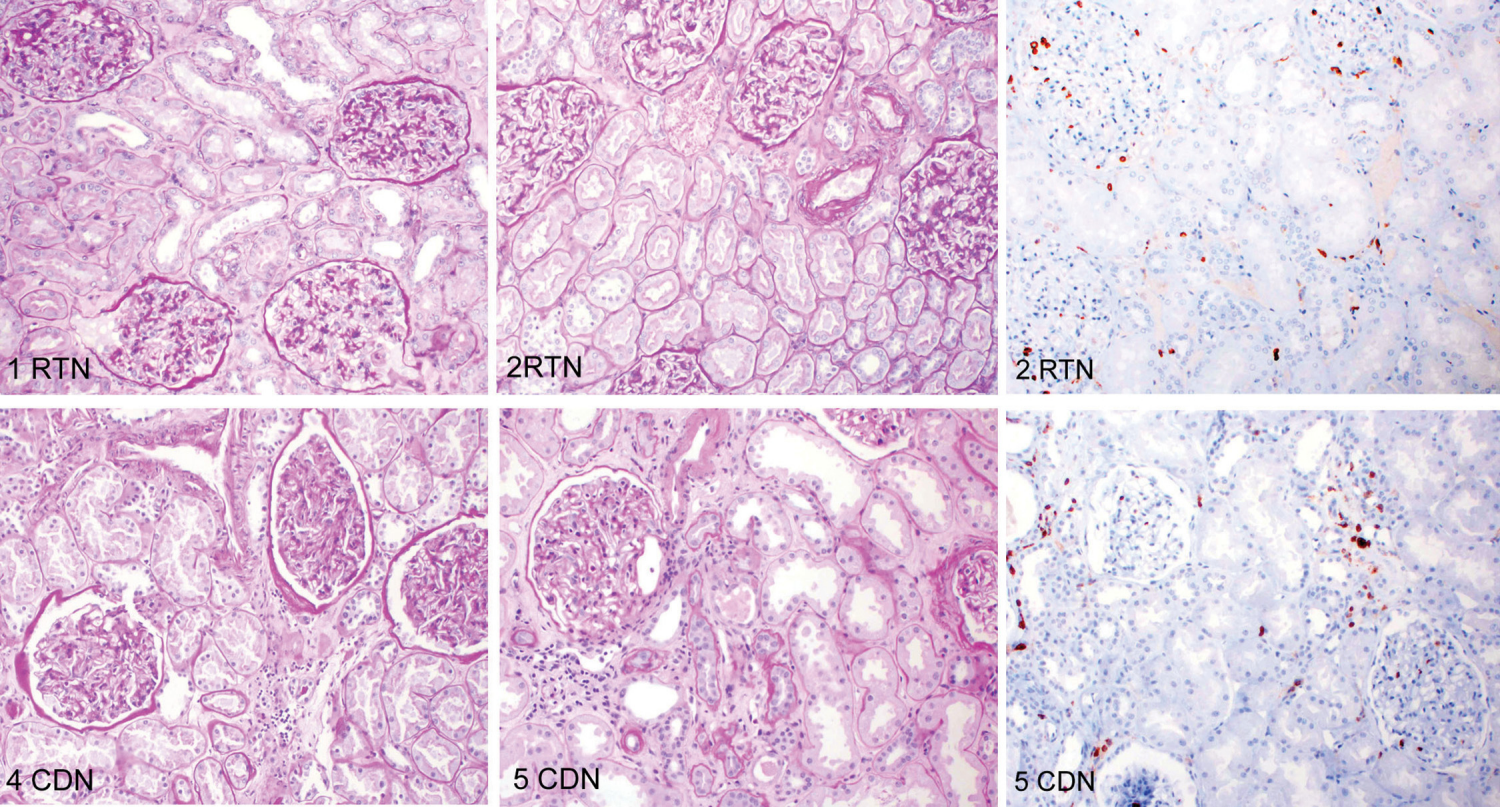
Immunohistological characterization. Sections from two RTN and CDN specimen adjacent to those used for FACS were stained for morphological evaluation with PAS and an antibody to CD45.

**Fig 9:**
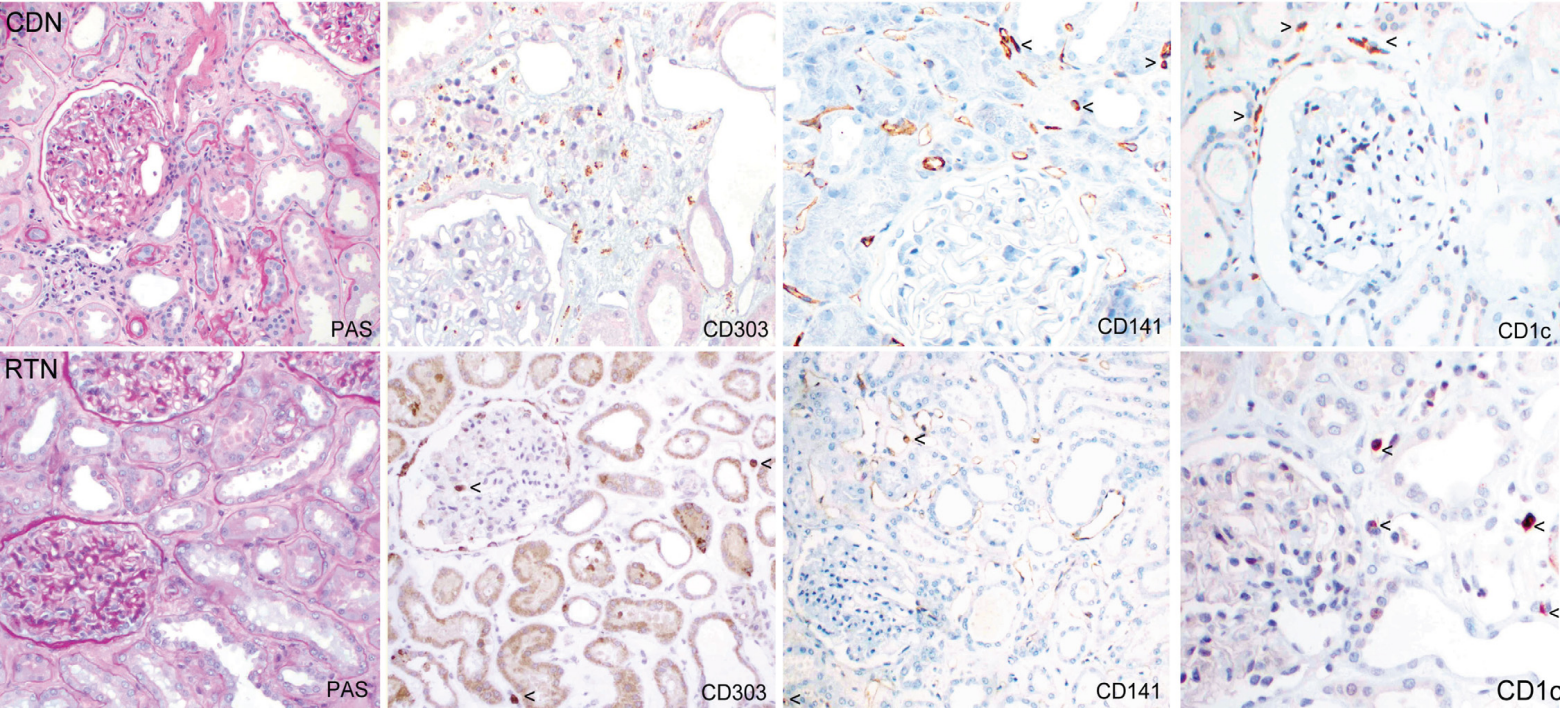
Localization of the DC subtypes in kidney cortex. Histological sections from CDN (case 5) and RTN (case 1) adjacent to those used for FACS were stained with CD303, CD141 and CD1c in order to localize the DC subtype and count their absolute number. CD303 expressing pDC (double staining with CD68 in brown) are rare in the renal cortex of the donor nephrectomy specimens (CDN) whereas CD141^+^ cells (arrows) were more common and CD1c^+^ cells (arrows) were easily detectable. There were three-to seven-fold more pDC (arrows) in the tumour nephrectomies (RTN) compared to the donor kidneys, but rarely any CD141 expressing cells (arrows).

Sequential kidney tissue sections taken from areas adjacent to those used for flow cytometry were stained with PAS, CD45 and CD68, commonly used in routine evaluation as well as with antibodies specific for CD303, CD141 and CD1c. Infiltrating leukocytes were compared in areas of fibrosis and tubular atrophy in sections from donor nephrectomy specimens (CDN) and tumor nephrectomies (RTN). As shown in Fig 8, overall numbers and pattern of infiltration appeared similar in sections from both groups using an antibody to the leukocyte common antigen (CD45). In both radical tumor nephrectomy specimens, CD303+ pDC were easily identified but could not be visualized in similar areas of specimens from normal kidney donors (Fig 9 and S2 Fig). Conversely, CD141+ cells were appreciably more abundant in the normal donor nephrectomy specimens than in the tumor nephrectomy specimens. There was no detectable difference number of CD1c+ interstitial cells (Table 5). This pattern of cellular distribution is also seen in areas of fibrosis with tubular atrophy, where CD303+ cells are abundant in kidneys from RTN but not CDN.

**Table 5:** Absolute count and relative percentage of Dendritic cell subtype in histological sections.

	1 RTN cell/cm^2^ (% DCs)	2 RTN cell/cm^2^ (% DCs)	3 RN cell/cm^2^ (% DCs)	4 CDN cell/cm^2^ (% DCs)	5 CDN cell/cm^2^ (% DCs)
Absolute pDC	135.8 (57.4%)	99.40 (52%)	79.6 (58%)	22.0 (7.5%)	9.6 (2.86%)
Absolute cDC1	38.2 (16.1%)	81.1 (42.5%)	55.8 (42%)	267 (89.7%)	294.8 (87.76%)
Absolute cDC2	190 (26.4%)	20.1 (5.3%)	NA	15.09 (2.8%)	57.9 (9.37%)

The cells were counted in sections taken from samples from the 5 nephrectomy specimens. The samples were 2.6 to 3.5 cm^2^ in area and the median number of cells of each subtypes counted was per specimens 145 (range 25–802).

## Discussion

Understanding how renal MPh networks execute their critical functions in homeostasis and inflammation requires detailed knowledge of the types of macrophages and DC involved and how they respond to injury. Much has been learned about the complexity in mice but little is known in humans. Here, we present a detailed description of MPh in the kidney and identify distinct populations of DC, monocytes, macrophages, each of which can further be resolved into well-defined subtypes. We identify a novel population of CD141 expressing macrophages and show that the renal infiltration with pDC commonly associated with injury does not occur in isolation but is part of a co-ordinated response affecting other renal MPh populations. We were able to show this because we *(i)* confined our analysis to morphologically normal portions of kidneys; *(ii)* combined it with a method of digestion that resulted in a single cells suspension; *(iii)* followed it by a ficoll density gradient centrifugation that enriched viable mononuclear cells whilst excluding cells debris, erythrocytes and polymorphnuclear cells and *(iv)* used a gating strategy that enabled us to characterize the whole MPh population. These data complement previous analysis of DC subsets in kidney with overt inflammation [30].

There is little difficulty in demonstrating differences in the abundance of pDC in injured organs because of their rarity in healthy tissues and because they express the same cell markers as in secondary lymphoid tissue. Quantifying the change in abundance of cDC presents greater challenges first because cDC1 and cDC2 are both common in organs under homeostatic conditions and after injury [36, 37]. Quantitation is further complicated by tissue specific differences in expression of the molecules traditionally used to identify them: for example in lymphoid tissue, cDC1 and cDC2 express CD8a and CD4a respectively whereas in other organs they express different tissue-specific markers, including CD103, Clec9 or CD11b. In leukocytes, CD141 (thrombomodulin) expression is restricted to cDC1 but it is also expressed on the microvascular endothelium, and so a multicolor strategy is essential for unequivocally identifying and enumerating CD141 expressing leukocytes.

In human blood, pDC, cDC1 and cDC2 are distinguished from other leukocytes by their respective expression of CD303, CD141 and CD1c and arise from circulating progenitors similar to those in mice [38, 39]. Our results demonstrate that antibodies specific for CD303 and CD1c also uniquely identify pDC and cDC2 resident in the kidney. Antibodies to CD141 recognised cDC1 cells whose identity was confirmed by forward and side scatter together with the co-expression of CD11c without CD11b, but its expression was not restricted to them. CD141 was also abundantly expressed by a previously unrecognized population of macrophage-like MPh that were larger and more granular and co-expressed CD11b but not CD11c. These could be monocyte-derived macrophages or even embryonic macrophage derived MPh as both cell types have been reported to express CD141 without being fully described. Alternatively, the CD141+ macrophages could be the equivalent of the F4/80^+^ dendritic cells found in mice kidney, [21, 40]. Further functional studies will be required to discriminate between these possibilities.

Monocytes were surprisingly common in the kidney specimens and could be readily segregated into the classical, intermediate and non-classical monocyte subsets found in blood. Although not typically regarded as interstitial cells under homeostatic conditions, their abundance in kidney argues against their being contaminants from blood, especially since the donor nephrectomies were perfused free of blood. The absence of macrophage markers CD68 and 25F9 in the renal monocytes/macrophages of kidneys precludes them from being recently emigrated monocytes in the early phase of differentiation into macrophages (or DC). These macrophage markers were detected in kidneys infiltrated with large numbers of pDC, suggesting a co-ordinate response to injury. Accordingly, it is likely that the monocytes detected by our studies are genuine interstitial monocytes analogous to those described in human skin [41].

In mice, non-classical monocytes in the kidney do not infiltrate the interstitium but remain within the vasculature adherent to the endothelial surface, where they act as sensors of injury [42]; and human non-classical monocytes display similar behavior when adoptively transferred into mice [43]. Potentially, these could resist perfusion and be the source of the non-classical macrophages found here. Non-activated classical monocytes have recently been identified in other tissues: in mice they traffic through the interstitium of lungs and carry antigens to the draining lymph nodes with evidence of activation or maturation [44]; and similar tissue monocytes have recently been identified in mouse and human skin [45] and could serve similar functions in the kidney. The kidneys with abundant pDC contained an additional population of CD14 positive cells that co-expressed the macrophage markers CD68 and 25F9. These molecules were not expressed by classical monocytes but only on CD14 cells that also expressed CD16. There was an inverse correlation between the abundance of CD14 and that of CD11b and CD11c in the CD68/25F9^+^ cells that strongly suggests they are recently emigrated classical monocytes undergoing maturation into macrophages.

Infiltration with pDC is a validated indicator of tissue injury in the kidney [27, 28]**.** Here we show that the signals responsible for pDC recruitment are induced by minor injury, even in the absence of overt inflammation detectable by light microscopy. The results also show that pDC infiltration is not an isolated event but is part of a more generalised response that affects most MPh populations. Thus pDC infiltration correlates with a striking proportionate reduction of cDC1 without a decrease of cDC2 population; and with a reduction of the CD141^+^ cells which is associated with the appearance nascent monocyte derived macrophages that express CD14 together with the mature macrophage markers CD68 and 25F9. The uniformity of the correlations preclude random variation as an explanation but instead indicate they constitute a co-ordinated stereotypic response to injury. The results are consistent with studies in mice [46] and may be analogous to those observed in of inflamed human skin and intestine [47, 48].

In summary, our study reveals the heterogeneity of MPh populations in the human kidney that we show contains the known subtypes of DC and monocyte together with a novel population of CD141^+^ cells with the physical properties of macrophages. We also show that minor injury, as indicated by pDC infiltration, induces a co-ordinated response that includes marked reduction in cDC1 cells and the appearance of nascent monocyte-derived macrophages. These results provide a novel insight into the role of DC and monocytes/macrophages in homeostasis and injury and have implications for understanding how renal inflammation influences this migration and influx of the innate immune cells.

## Supporting Information

S1 Fig Isotype staining. (EPS)

S2 Fig Ratio CD1c/10CD303. (EPS)
